# 4-Amino-3-methyl­benzoic acid–1,2-bis­(4-pyrid­yl)ethane (1/1)

**DOI:** 10.1107/S1600536811005381

**Published:** 2011-02-19

**Authors:** Shie Fu Lush, Chong Wei Chen, Chieh Yang, Fwu Ming Shen

**Affiliations:** a Department of General Education Center, Yuanpei University, HsinChu 30015, Taiwan; b Department of Medical Laboratory Science Biotechnology, Yuanpei University, HsinChu 30015, Taiwan; c Department of Biotechnology, Yuanpei University, HsinChu 30015, Taiwan

## Abstract

In the crystal structure of the title 1:1 adduct, C_12_H_12_N_2_·C_8_H_9_NO_2_, the 4-amino-3-methyl­benzoic acid mol­ecules and 1,2-bis­(4-pyrid­yl)ethane mol­ecules are linked by inter­molecular O—H⋯N, N—H⋯O and N—H⋯N hydrogen bonds, forming a two-dimensional supra­molecular network parallel to (001). In the 1,2-bis­(4-pyrid­yl)ethane mol­ecule, the two pyridine rings are twisted to each other by a dihedral angle of 12.12 (8)°. The non-H atoms of the 4-amino-3-methyl­benzoic acid mol­ecule are almost coplanar, the maximum atomic deviation being 0.029 (1) Å. Weak C—H⋯π inter­actions are present in the crystal structure.

## Related literature

For related structures, see: Bowes *et al.* (2003 ▶); Ferguson *et al.* (1999 ▶); Shen & Lush (2010 ▶). For hydrogen-bond motifs, see: Etter *et al.* (1990 ▶).
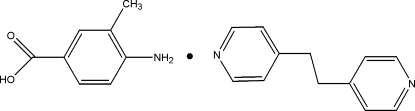


## Experimental

### 

#### Crystal data

C_12_H_12_N_2_·C_8_H_9_NO_2_
*M* *_r_* = 335.40
Monoclinic, 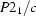

*a* = 8.0695 (3) Å
*b* = 13.0677 (5) Å
*c* = 17.6138 (10) Å
β = 99.501 (5)°
*V* = 1831.89 (15) Å^3^
*Z* = 4
Mo *K*α radiation
μ = 0.08 mm^−1^
*T* = 297 K
0.60 × 0.18 × 0.12 mm

#### Data collection

Oxford Diffraction Gemini-S CCD diffractometer
Absorption correction: multi-scan (*CrysAlis PRO*; Oxford Diffraction, 2009 ▶) *T*_min_ = 0.919, *T*_max_ = 1.000
8821 measured reflections
4277 independent reflections
1870 reflections with *I* > 2σ(*I*)
*R*_int_ = 0.025

#### Refinement

*R*[*F* ^2^ > 2σ(*F* ^2^)] = 0.044
*wR*(*F* ^2^) = 0.082
*S* = 1.03
4277 reflections
232 parameters
3 restraints
H atoms treated by a mixture of independent and constrained refinement
Δρ_max_ = 0.15 e Å^−3^
Δρ_min_ = −0.22 e Å^−3^

### 

Data collection: *CrysAlis CCD* (Oxford Diffraction, 2008 ▶); cell refinement: *CrysAlis RED* (Oxford Diffraction, 2008 ▶); data reduction: *CrysAlis RED*; program(s) used to solve structure: *SHELXS97* (Sheldrick, 2008 ▶); program(s) used to refine structure: *SHELXL97* (Sheldrick, 2008 ▶); molecular graphics: *PLATON* (Spek, 2009 ▶); software used to prepare material for publication: *PLATON*.

## Supplementary Material

Crystal structure: contains datablocks global, I. DOI: 10.1107/S1600536811005381/xu5156sup1.cif

Structure factors: contains datablocks I. DOI: 10.1107/S1600536811005381/xu5156Isup2.hkl

Additional supplementary materials:  crystallographic information; 3D view; checkCIF report

## Figures and Tables

**Table 1:** Hydrogen-bond geometry (Å, °) *Cg* is the centroid of the C2–C7 ring.

*D*—H⋯*A*	*D*—H	H⋯*A*	*D*⋯*A*	*D*—H⋯*A*
O1—H1*A*⋯N2	0.821 (9)	1.826 (11)	2.6407 (18)	171.6 (15)
N1—H1*B*⋯O2^i^	0.860 (11)	2.113 (12)	2.951 (2)	164.5 (14)
N1—H1*C*⋯N3^ii^	0.860 (7)	2.288 (9)	3.084 (2)	153.8 (14)
C12—H12*A*⋯*Cg*^iii^	0.93	2.76	3.540 (2)	141

Symmetry codes: (i) 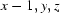; (ii) 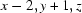; (iii) 
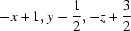
.

**Fig. 1.**
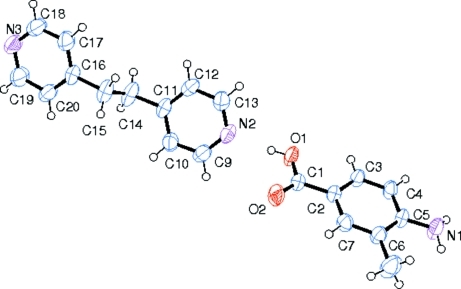
View of the title compound. Displacement ellipsoids for non-H atoms are drawn at the 50% probability level.

**Fig. 2.**
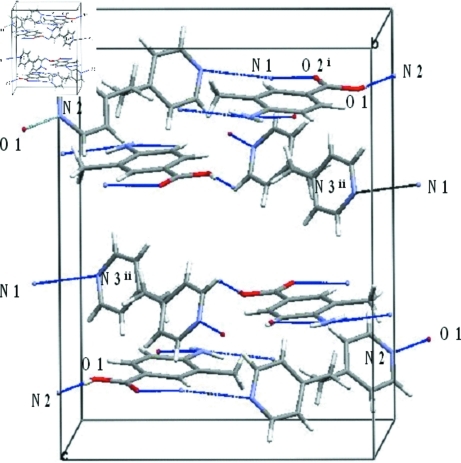
The molecular packing for the title compound. Hydrogen bonds are shown as dashed lines.

## supplementary crystallographic information

### Comment

The 1,2-bis(4-pyridyl)ethane is a versatile building block for the purposes of crystal engineering. Each of the pyridyl N atoms acts as a hydrogen bond acceptor, forming linear hydrogen associations (Ferguson *et al.*, 1999). Other structures related with 1,2-bis(4-pyridyl)ethane and Lewis acid were reported by Bowes *et al.* (2003) and Shen & Lush (2010). We present here the crystal structure of the 4-amino-3-methybenzoic acid and 1,2-bis(4-pyridyl)ethane 1:1 adduct.

The structure of the title compound comprises 4-amino-3-methybenzoic acid molecule and 1,2-bis(4-pyridyl)ethane molecule, with no proton transfer. In the structure, the molecules associate 4-amino-3-methybenzoic acid and 1,2-bis(4-pyridyl)ethane *via* carboxylic and pyridine group O—H···N [O···N 2.640 (18) Å] C^2^_2_(19) (Etter *et al.*, 1990), forming linear hydrogen bonding parallel to [0 0 1], further connect a two dimensional network *via* amine and carboxylic N—H···O and N—H···N [2.951 (2) and 3.084 (2) Å], respectively. Furthermore, C—H···π ring stacking interaction is present in the structure. The distance between C12—H(12 A)···*Cg*3 ^iii^(C2—C7) is 3.540 (2) Å [symmetry code: (iii) = 1-*X*,-1/2+*Y*,3/2-*Z*].

### Experimental

The 4-amino-3-methybenzoic acid (151 mg, 1 mmol) and 1,2-bis(4-pyridyl)ethane (184 mg, 1 mmol) were dissolved in 20 ml methanol, the solution was refluxed for 30 min. The filtered solution was transferred to a 25 ml tube, at room temperature colorless crystals were formed after several days (yield 59.12%).

### Refinement

Amino H atoms were located in a difference Fourier map and were refined with the distance constraints of N—H = 0.860±0.001 Å. Other H atoms were positioned geometrically with C—H = 0.93-0.97 Å, and refined using a riding model, U_iso_(H) = 1.5U_eq_(C) for methyl H atoms and 1.2U_eq_(C) for the others.

### Crystal data

**Table:**

C_12_H_12_N_2_·C_8_H_9_NO_2_	*F*(000) = 712
*M**_r_* = 335.40	*D*_x_ = 1.216 Mg m^−^^3^
Monoclinic, *P*2_1_/*c*	Mo *K*α radiation, λ = 0.71073 Å
Hall symbol: -P 2ybc	Cell parameters from 2506 reflections
*a* = 8.0695 (3) Å	θ = 3.0–29.0°
*b* = 13.0677 (5) Å	µ = 0.08 mm^−^^1^
*c* = 17.6138 (10) Å	*T* = 297 K
β = 99.501 (5)°	Parallelepiped, colorless
*V* = 1831.89 (15) Å^3^	0.60 × 0.18 × 0.12 mm
*Z* = 4	

### Data collection

**Table:**

Oxford Diffraction Gemini-S CCD diffractometer	4277 independent reflections
Radiation source: fine-focus sealed tube	1870 reflections with *I* > 2σ(*I*)
graphite	*R*_int_ = 0.025
ω scans	θ_max_ = 29.1°, θ_min_ = 3.0°
Absorption correction: multi-scan (*CrysAlis PRO*; Oxford Diffraction, 2009)	*h* = −10→10
*T*_min_ = 0.919, *T*_max_ = 1.000	*k* = −17→16
8821 measured reflections	*l* = −24→20

### Refinement

**Table:**

Refinement on *F*^2^	Primary atom site location: structure-invariant direct methods
Least-squares matrix: full	Secondary atom site location: difference Fourier map
*R*[*F*^2^ > 2σ(*F*^2^)] = 0.044	Hydrogen site location: inferred from neighbouring sites
*wR*(*F*^2^) = 0.082	H atoms treated by a mixture of independent and constrained refinement
*S* = 1.03	*w* = 1/[σ^2^(*F*_o_^2^) + (0.025*P*)^2^] where *P* = (*F*_o_^2^ + 2*F*_c_^2^)/3
4277 reflections	(Δ/σ)_max_ < 0.001
232 parameters	Δρ_max_ = 0.15 e Å^−^^3^
3 restraints	Δρ_min_ = −0.22 e Å^−^^3^

### Special details

**Table:**

Geometry. Bond distances, angles *etc*. have been calculated using the rounded fractional coordinates. All su's are estimated from the variances of the (full) variance-covariance matrix. The cell e.s.d.'s are taken into account in the estimation of distances, angles and torsion angles
Refinement. Refinement on *F*^2^ for ALL reflections except those flagged by the user for potential systematic errors. Weighted *R*-factors *wR* and all goodnesses of fit *S* are based on *F*^2^, conventional *R*-factors *R* are based on *F*, with *F* set to zero for negative *F*^2^. The observed criterion of *F*^2^ > σ(*F*^2^) is used only for calculating -*R*-factor-obs *etc*. and is not relevant to the choice of reflections for refinement. *R*-factors based on *F*^2^ are statistically about twice as large as those based on *F*, and *R*-factors based on ALL data will be even larger.

### Fractional atomic coordinates and isotropic or equivalent isotropic displacement parameters (Å^2^)

**Table:**

	*x*	*y*	*z*	*U*_iso_*/*U*_eq_	
N2	0.63037 (17)	0.47008 (10)	0.62737 (9)	0.0552 (6)	
N3	1.58754 (19)	0.08351 (11)	0.60868 (10)	0.0692 (7)	
C9	0.7078 (2)	0.47692 (12)	0.56699 (11)	0.0553 (7)	
C10	0.8385 (2)	0.41502 (12)	0.55562 (10)	0.0521 (7)	
C11	0.8957 (2)	0.34059 (12)	0.60903 (11)	0.0482 (7)	
C12	0.8172 (2)	0.33448 (13)	0.67191 (11)	0.0650 (8)	
C13	0.6872 (2)	0.39953 (15)	0.67901 (11)	0.0683 (8)	
C14	1.0359 (2)	0.26912 (12)	0.59857 (12)	0.0705 (8)	
C15	1.2064 (2)	0.30811 (12)	0.62896 (11)	0.0660 (7)	
C16	1.3425 (2)	0.23093 (12)	0.62204 (11)	0.0506 (7)	
C17	1.4071 (2)	0.16866 (14)	0.68217 (11)	0.0639 (8)	
C18	1.5274 (2)	0.09729 (14)	0.67281 (12)	0.0710 (8)	
C19	1.5251 (2)	0.14425 (15)	0.55151 (12)	0.0808 (9)	
C20	1.4039 (2)	0.21762 (13)	0.55528 (11)	0.0686 (8)	
O1	0.34436 (14)	0.56570 (8)	0.63298 (7)	0.0542 (5)	
O2	0.47629 (14)	0.71109 (8)	0.61635 (7)	0.0664 (5)	
N1	−0.25914 (19)	0.86901 (13)	0.64324 (10)	0.0628 (7)	
C1	0.34970 (19)	0.66628 (12)	0.62564 (9)	0.0399 (4)	
C2	0.18870 (19)	0.71739 (11)	0.63001 (9)	0.0399 (4)	
C3	0.04752 (18)	0.66405 (11)	0.64278 (9)	0.0427 (6)	
C4	−0.09994 (19)	0.71458 (11)	0.64585 (9)	0.0451 (6)	
C5	−0.11212 (19)	0.82069 (12)	0.63671 (9)	0.0421 (6)	
C6	0.0301 (2)	0.87564 (11)	0.62414 (9)	0.0465 (6)	
C7	0.17592 (19)	0.82300 (11)	0.62061 (9)	0.0461 (6)	
C8	0.0217 (2)	0.99081 (11)	0.61582 (12)	0.0818 (9)	
H9A	0.67140	0.52660	0.53020	0.0660*	
H10A	0.88840	0.42320	0.51200	0.0620*	
H12A	0.85190	0.28610	0.71000	0.0780*	
H13A	0.63640	0.39370	0.72250	0.0820*	
H14A	1.01960	0.20490	0.62390	0.0850*	
H14B	1.02920	0.25500	0.54410	0.0850*	
H15A	1.21180	0.32640	0.68270	0.0790*	
H15B	1.22680	0.36960	0.60110	0.0790*	
H17A	1.36980	0.17460	0.72920	0.0770*	
H18A	1.56900	0.05610	0.71470	0.0850*	
H19A	1.56560	0.13720	0.50530	0.0970*	
H20A	1.36450	0.25770	0.51250	0.0820*	
H1A	0.4380 (9)	0.5416 (12)	0.6323 (10)	0.082 (7)*	
H1B	−0.3449 (12)	0.8295 (10)	0.6418 (10)	0.075 (7)*	
H1C	−0.2717 (19)	0.9312 (4)	0.6273 (8)	0.055 (6)*	
H3A	0.05290	0.59340	0.64930	0.0510*	
H4A	−0.19370	0.67760	0.65420	0.0540*	
H7A	0.26980	0.85930	0.61160	0.0550*	
H8A	0.12810	1.01620	0.60650	0.1230*	
H8B	−0.00420	1.02060	0.66230	0.1230*	
H8C	−0.06420	1.00870	0.57340	0.1230*	

### Atomic displacement parameters (Å^2^)

**Table:**

	*U*^11^	*U*^22^	*U*^33^	*U*^12^	*U*^13^	*U*^23^
N2	0.0437 (9)	0.0556 (9)	0.0657 (11)	0.0178 (7)	0.0069 (9)	0.0008 (8)
N3	0.0678 (11)	0.0797 (11)	0.0613 (12)	0.0363 (9)	0.0146 (9)	0.0079 (10)
C9	0.0509 (12)	0.0498 (10)	0.0631 (14)	0.0097 (9)	0.0032 (10)	0.0069 (10)
C10	0.0460 (11)	0.0559 (11)	0.0560 (13)	0.0066 (9)	0.0135 (9)	−0.0026 (10)
C11	0.0385 (10)	0.0426 (11)	0.0617 (13)	0.0093 (8)	0.0030 (10)	−0.0118 (10)
C12	0.0609 (13)	0.0670 (12)	0.0668 (14)	0.0282 (10)	0.0097 (11)	0.0173 (11)
C13	0.0588 (13)	0.0887 (14)	0.0608 (14)	0.0230 (11)	0.0196 (10)	0.0105 (12)
C14	0.0474 (12)	0.0566 (11)	0.1053 (17)	0.0179 (10)	0.0062 (11)	−0.0171 (11)
C15	0.0442 (11)	0.0605 (11)	0.0922 (16)	0.0139 (10)	0.0082 (11)	−0.0143 (11)
C16	0.0388 (11)	0.0516 (11)	0.0611 (14)	0.0114 (9)	0.0077 (10)	−0.0091 (10)
C17	0.0597 (13)	0.0812 (13)	0.0535 (14)	0.0216 (11)	0.0173 (10)	−0.0002 (11)
C18	0.0713 (14)	0.0834 (15)	0.0572 (15)	0.0318 (11)	0.0071 (12)	0.0163 (11)
C19	0.0877 (16)	0.1022 (16)	0.0580 (15)	0.0442 (14)	0.0285 (12)	0.0109 (13)
C20	0.0699 (14)	0.0747 (13)	0.0617 (15)	0.0361 (11)	0.0127 (11)	0.0150 (11)
O1	0.0387 (8)	0.0491 (7)	0.0761 (9)	0.0153 (6)	0.0130 (7)	−0.0007 (6)
O2	0.0306 (7)	0.0689 (8)	0.1025 (10)	−0.0004 (6)	0.0192 (7)	0.0135 (7)
N1	0.0418 (10)	0.0476 (10)	0.1012 (14)	0.0112 (9)	0.0184 (10)	−0.0012 (10)
C1	0.0324 (7)	0.0430 (7)	0.0442 (7)	0.0040 (5)	0.0058 (5)	−0.0032 (6)
C2	0.0324 (7)	0.0430 (7)	0.0442 (7)	0.0040 (5)	0.0058 (5)	−0.0032 (6)
C3	0.0354 (10)	0.0332 (8)	0.0604 (12)	0.0031 (8)	0.0109 (8)	−0.0035 (8)
C4	0.0313 (10)	0.0403 (10)	0.0651 (13)	−0.0011 (8)	0.0123 (8)	−0.0038 (8)
C5	0.0316 (9)	0.0430 (10)	0.0519 (12)	0.0085 (8)	0.0072 (8)	−0.0072 (9)
C6	0.0411 (11)	0.0378 (9)	0.0606 (13)	0.0039 (9)	0.0088 (9)	−0.0002 (9)
C7	0.0354 (10)	0.0444 (10)	0.0594 (12)	−0.0042 (8)	0.0109 (9)	0.0003 (9)
C8	0.0647 (14)	0.0445 (11)	0.138 (2)	0.0047 (9)	0.0217 (13)	0.0091 (11)

### Geometric parameters (Å, °)

**Table:**

O1—C1	1.3221 (19)	C13—H13A	0.9300
O2—C1	1.2118 (19)	C14—H14A	0.9700
O1—H1A	0.821 (9)	C14—H14B	0.9700
N2—C13	1.323 (2)	C15—H15B	0.9700
N2—C9	1.322 (2)	C15—H15A	0.9700
N3—C18	1.313 (3)	C17—H17A	0.9300
N3—C19	1.315 (3)	C18—H18A	0.9300
N1—C5	1.366 (2)	C19—H19A	0.9300
N1—H1C	0.860 (7)	C20—H20A	0.9300
N1—H1B	0.860 (11)	C1—C2	1.474 (2)
C9—C10	1.370 (2)	C2—C3	1.385 (2)
C10—C11	1.379 (2)	C2—C7	1.392 (2)
C11—C12	1.366 (3)	C3—C4	1.370 (2)
C11—C14	1.502 (2)	C4—C5	1.398 (2)
C12—C13	1.372 (2)	C5—C6	1.402 (2)
C14—C15	1.482 (2)	C6—C7	1.373 (2)
C15—C16	1.511 (2)	C6—C8	1.513 (2)
C16—C17	1.369 (3)	C3—H3A	0.9300
C16—C20	1.360 (3)	C4—H4A	0.9300
C17—C18	1.376 (2)	C7—H7A	0.9300
C19—C20	1.379 (2)	C8—H8A	0.9600
C9—H9A	0.9300	C8—H8B	0.9600
C10—H10A	0.9300	C8—H8C	0.9600
C12—H12A	0.9300		
			
C1—O1—H1A	109.6 (10)	H15A—C15—H15B	108.00
C9—N2—C13	116.35 (14)	C14—C15—H15A	109.00
C18—N3—C19	115.24 (16)	C16—C17—H17A	120.00
H1B—N1—H1C	120.4 (14)	C18—C17—H17A	120.00
C5—N1—H1B	115.2 (8)	N3—C18—H18A	118.00
C5—N1—H1C	117.8 (10)	C17—C18—H18A	118.00
N2—C9—C10	123.71 (16)	C20—C19—H19A	118.00
C9—C10—C11	119.73 (16)	N3—C19—H19A	118.00
C10—C11—C12	116.53 (15)	C16—C20—H20A	120.00
C10—C11—C14	121.95 (16)	C19—C20—H20A	120.00
C12—C11—C14	121.52 (16)	O2—C1—C2	123.92 (14)
C11—C12—C13	120.11 (17)	O1—C1—O2	122.37 (14)
N2—C13—C12	123.55 (17)	O1—C1—C2	113.71 (13)
C11—C14—C15	114.53 (14)	C1—C2—C7	119.45 (14)
C14—C15—C16	112.66 (14)	C3—C2—C7	118.13 (14)
C15—C16—C17	121.57 (16)	C1—C2—C3	122.42 (13)
C15—C16—C20	121.90 (16)	C2—C3—C4	120.46 (14)
C17—C16—C20	116.51 (16)	C3—C4—C5	121.25 (14)
C16—C17—C18	119.77 (17)	N1—C5—C4	119.65 (15)
N3—C18—C17	124.36 (18)	N1—C5—C6	121.45 (15)
N3—C19—C20	124.59 (18)	C4—C5—C6	118.85 (14)
C16—C20—C19	119.53 (17)	C5—C6—C8	120.01 (14)
C10—C9—H9A	118.00	C7—C6—C8	121.27 (14)
N2—C9—H9A	118.00	C5—C6—C7	118.72 (14)
C9—C10—H10A	120.00	C2—C7—C6	122.58 (14)
C11—C10—H10A	120.00	C2—C3—H3A	120.00
C11—C12—H12A	120.00	C4—C3—H3A	120.00
C13—C12—H12A	120.00	C3—C4—H4A	119.00
C12—C13—H13A	118.00	C5—C4—H4A	119.00
N2—C13—H13A	118.00	C2—C7—H7A	119.00
C11—C14—H14A	109.00	C6—C7—H7A	119.00
C11—C14—H14B	109.00	C6—C8—H8A	109.00
H14A—C14—H14B	108.00	C6—C8—H8B	109.00
C15—C14—H14A	109.00	C6—C8—H8C	109.00
C15—C14—H14B	109.00	H8A—C8—H8B	109.00
C16—C15—H15A	109.00	H8A—C8—H8C	110.00
C14—C15—H15B	109.00	H8B—C8—H8C	110.00
C16—C15—H15B	109.00		
			
C13—N2—C9—C10	0.9 (3)	C16—C17—C18—N3	0.0 (3)
C9—N2—C13—C12	−1.0 (3)	N3—C19—C20—C16	0.6 (3)
C19—N3—C18—C17	0.4 (3)	O1—C1—C2—C3	1.7 (2)
C18—N3—C19—C20	−0.7 (3)	O1—C1—C2—C7	−177.92 (14)
N2—C9—C10—C11	0.0 (3)	O2—C1—C2—C3	−178.20 (16)
C9—C10—C11—C12	−0.9 (2)	O2—C1—C2—C7	2.2 (2)
C9—C10—C11—C14	178.60 (16)	C1—C2—C3—C4	−179.52 (15)
C10—C11—C12—C13	0.8 (3)	C7—C2—C3—C4	0.1 (2)
C14—C11—C12—C13	−178.66 (16)	C1—C2—C7—C6	−179.85 (15)
C10—C11—C14—C15	87.8 (2)	C3—C2—C7—C6	0.5 (2)
C12—C11—C14—C15	−92.8 (2)	C2—C3—C4—C5	−0.4 (2)
C11—C12—C13—N2	0.1 (3)	C3—C4—C5—N1	−177.34 (16)
C11—C14—C15—C16	176.12 (16)	C3—C4—C5—C6	0.0 (2)
C14—C15—C16—C17	−96.3 (2)	N1—C5—C6—C7	177.88 (16)
C14—C15—C16—C20	81.9 (2)	N1—C5—C6—C8	−1.3 (2)
C15—C16—C17—C18	178.08 (16)	C4—C5—C6—C7	0.6 (2)
C20—C16—C17—C18	−0.1 (2)	C4—C5—C6—C8	−178.64 (15)
C15—C16—C20—C19	−178.34 (16)	C5—C6—C7—C2	−0.9 (2)
C17—C16—C20—C19	−0.1 (2)	C8—C6—C7—C2	178.32 (16)

### Hydrogen-bond geometry (Å, °)

**Table:**

Cg is the centroid of the C2–C7 ring.

**Table:**

*D*—H···*A*	*D*—H	H···*A*	*D*···*A*	*D*—H···*A*
O1—H1A···N2	0.821 (9)	1.826 (11)	2.6407 (18)	171.6 (15)
N1—H1B···O2^i^	0.860 (11)	2.113 (12)	2.951 (2)	164.5 (14)
N1—H1C···N3^ii^	0.860 (7)	2.288 (9)	3.084 (2)	153.8 (14)
C12—H12A···Cg^iii^	0.93	2.76	3.540 (2)	141

Symmetry codes: (i) *x*−1, *y*, *z*; (ii) *x*−2, *y*+1, *z*; (iii) −*x*+1, *y*−1/2, −*z*+3/2.
